# Root growth and spatial distribution characteristics for seedlings raised in substrate and transplanted cotton

**DOI:** 10.1371/journal.pone.0190032

**Published:** 2017-12-22

**Authors:** Xiaoyu Zhi, Yingchun Han, Yabing Li, Guoping Wang, Lu Feng, Beifang Yang, Zhengyi Fan, Yaping Lei, Wenli Du, Shuchun Mao

**Affiliations:** Institute of Cotton Research of the Chinese Academy of Agricultural Sciences/State Key Laboratory of Cotton Biology, Anyang, Henan, P.R. China; University of Maryland Center for Environmental Science, UNITED STATES

## Abstract

In this study, transplanting cotton seedlings grown in artificial substrate is considered due to recent increased interest in cotton planting labor saving approaches. The nursery methods used for growing cotton seedlings affect root growth. However, the underlying functional responses of root growth to variations in cotton seedling transplanting methods are poorly understood. We assessed the responses of cotton (*Gossypium hirsutum* L.) roots to different planting methods by conducting cotton field experiments in 2012 and 2013. A one-factor random block design was used with three replications and three different cotton planting patterns (substrate seedling transplanted cotton (SSTC), soil-cube seedling transplanted cotton (ScSTC) and directly sown cotton (DSC). The distributions and variances of the root area density (RAD) and root length density (RLD) at different cotton growing stages and several yield components were determined. Overall, the following results were observed: 1) The RAD and RLD were greatest near the plants (a horizontal distance of 0 cm) but were lower at W20 and W40 cm in the absence of film mulching than at E20 and E40 cm with film mulching. 2) The roots were confined to shallow depths (20–40 cm), and the root depths of SSTC and DSC were greater than the root depths of ScSTC. 3) Strong root growth was observed in the SSTC at the cotton flowering and boll setting stages. In addition, early onset root growth occurred in the ScSTC, and vigorous root growth occurred throughout all cotton growth stages in DSC. 4) The SSTC plants had more lateral roots with higher root biomass (RB) than the ScSTC, which resulted in higher cotton yields. However, the early onset root growth in the ScSTC resulted in greater pre-frost seed cotton (PFSC) yields. These results can be used to infer how cotton roots are distributed in soils and capture nutrients.

## Introduction

Cotton (*Gossypium hirsutum* L.) is one of the most important fiber crops in the world. China is the largest producer and consumer of cotton [1–4] and has three main cotton growing regions. In the Yellow River and Yangtze River Valleys, most of the cotton is relay-intercropped with grain, which results in greater sunlight interception, multiple crop indexes and economic benefits [5–8].

The competition of relay intercropped plants for water, nutrients and sunshine is intense, particularly between cotton and wheat (*Triticumaestivum Linn*.) when cotton is sown before wheat being harvested [9]. This competition delays cotton growth and reduces cotton yield. But transplanting cotton seedlings is an effective method for preventing or alleviating the negative effects of relay intercropping on both crops [10–13] and can eliminate the early season chilling stress before transplanting [12, 14–15]. Moreover, columned soil blocks made from soil and organic fertilizer are commonly used in seedling nurseries [3, 16], which can reduce the costs of growing cotton seedlings and the incidence of disease or insect pests and improve cotton establishment [17]. However, seedling transplanting, which is an intensive cotton farming practice, requires a great deal of labor, especially for making soil-cubes to establish seedlings in a nursery [18–19]. Thus, although transplanting seedlings is helpful, producers face significant challenges due to the labor shortage caused by rapid urbanization and the transfer of labor from rural areas to cities or towns [20–21]. To resolve the problem of limited agricultural labor in China, highly efficient and simplified seedling transplanting methods are needed.

Transplanting seedlings from vermiculite substrates, which are widely used in crop seedlings production [22–26], can reduce the amount and intensity of manual labor required by eliminating the need to make soil cubes to facilitate industrial cotton seedling growth and mechanized transplanting [10, 19]. Additionally, local farmers have used substrate seedling transplanting for growing cotton seedlings in nurseries. However, the performances of cotton seedlings transplanted from substrates and using conventional techniques and cotton sown directly in the field have not been documented yet.

Roots provide an important link between soils and plants [27–28]. Furthermore, root systems have important physiological and biological functions for crop growth and yield [29–30]. Seed cotton yields and lint quality depend on the health status of cotton roots, moreover cotton root growth and development are significantly influenced by soil conditions, for instance soil texture and structure in that Ahmadi et al. found most potato (cv. Folva) roots accumulated in the surface layers of coarse sand as compared to the other soil types, yet higher root density being found in loamy sand than sandy loam and coarse sand [31–32]. Thus, it is important to understand root development and distribution when studying cotton seedling performance using different seedling growth techniques. Because they are generally hidden, it can be more challenging to analyze plant root systems than above ground plant components; however, plant root distributions can be accurately obtained by washing the soil off the roots [33]. Additionally, studies of root length density (RLD) and root area density (RAD) are preferable to studies of root weight density for delineating root distributions and characterizing root systems, as RLD and RAD are more scientifically meaningful for evaluation of plant root function in that the uptake of water and nutrients by roots depends on the total soil area with which the roots have contact [34–38]. However, little is known of how RLD and RAD differ between different seedling transplanting methods.

The main objective of this study was to investigate the differences in the spatial distributions of roots between directly sown cotton, cotton seedlings transplanted from substrate, and cotton seedlings transplanted from soil cubes. In addition, we aim to describe cotton root growth and two-dimensional cotton root distributions at different growth stages and determine the effects of seedling transplanting on cotton seed and other yield components.

## Materials and methods

### Experiment site

The field experiment was conducted in 2012 and 2013 at the experimental farm of the Institute of Cotton Research of the Chinese Academy of Agricultural Sciences in Anyang, Henan, China (36° 06 'N, 114° 21' E). The soil at the field site has a loam texture with total N, P and K contents of 820.0, 30.6 and 285.5 mg kg^-1^, respectively. Meteorological information was obtained from a station at the experimental site. The average temperatures from April to October were 22.3°C in 2012 and 21.6°C in 2013; sunshine duration was 1092 h in 2012 and 1157 h in 2013; active accumulated temperatures (≥15°C) were 4338°C in 2012 and 3998°C in 2013; and the total rainfall was 408 mm in 2012 and 480 mm in 2013.

#### Treatments, experimental design and management

In this study, a single factor experiment with three planting methods substrate seedling transplanted cotton (SSTC), soil-cube seedling transplanted cotton (ScSTC) and directly sown cotton (DSC) arranged in a completely randomized block design with three replicates in cotton. For all treatments, transgenic insect resistant *Bt* (*Bacillus thuringiensis*) cotton (*Gossipium hirsutium* L.) cultivar CRI46 was planted at a population density of 45000 plants ha^-1^. The plot was 6.4 m wide and 8 m long and consisted of 8 cotton rows with inter-row spacing of 80 cm. To enhance the reproducibility of our results, the laboratory protocols were available online at: dx.doi.org/10.17504/protocols.io.js4cngw

The substrate used in the substrate seedling transplanting system has a national patent certificate and was provided by the Institute of Cotton Research of the Chinese Academy of Agricultural Sciences. The patented substrate contains vermiculite, organic fertilizer and minerals that are safe for the environment, humans and livestock. Additionally, better aeration performance and water-holding capacity were observed. Based on local agronomic practices, on March 29–30 in both years, two cotton seeds were sown in each cavity of trays containing 100 cavities (60 cm*33 cm*5 cm) and placed in greenhouse-like huts with lowest temperature of 6°C and highest temperature of 20°C which were about 5°C higher than outside air to provide favorable conditions for seedling emergence and growth. According to the experimental design in both years, seedlings were transplanted on April 30 into 10-cm-deep holes that were dug with a hand machine which was homemade.

In the soil-cube seedling transplanting system, for raising seedlings, columned soil blocks (4–6 cm in diameter and 8–12 cm high), made of soil and fertilizer (N:15%, P_2_O_5_:15%, K_2_O:15%) with corresponding sizes of molds, were prepared in mid-March before planting. Next, the seeded soil cubes were placed neatly in seedling beds (10 cm deep and 2 m wide) in a greenhouse-like hut. One seed was sown in each block as soon as watering occurred on March 29–30 in both years. Blocks with seedlings were transplanted into 10-cm-deep holes manually in the experimental plots on April 30 according to the experiment design.

Cotton was planted on April 17 in the conventional planting and direct sowing systems with plastic mulching. The cotton seeds were covered with moist soil in all systems and mulched with wide plastic film (6 μm thick and 120 cm wide) that was held in place by burying its edges with soil along two rows. After emergence, holes were cut in the film to allow the seedlings to emerge. All plots were thinned to the desired plant density by leaving the most vigorous plants at the 2-true leaves stage.

In both experiments in each year, the land was ploughed and irrigated in early spring before sowing. In all plots, 225 kg ha^-1^ N, 150 kg ha^-1^ P_2_O_5_ and 225 kg ha^-1^ K_2_O was applied. All of the fertilizers were broadcasted evenly across the soil and incorporated into the top 20 cm of the soil before sowing. Approximately 45 mm of supplemental water (per irrigation event) was added by flooding the furrows and according to the recommendations of local agronomists. Other field management strategies were used based on local agronomic practices. Weeds were manually controlled before plastic mulch being used and pesticides were used to control insects and diseases.

### Data collection

#### Root distribution studies

**Fig 1:**
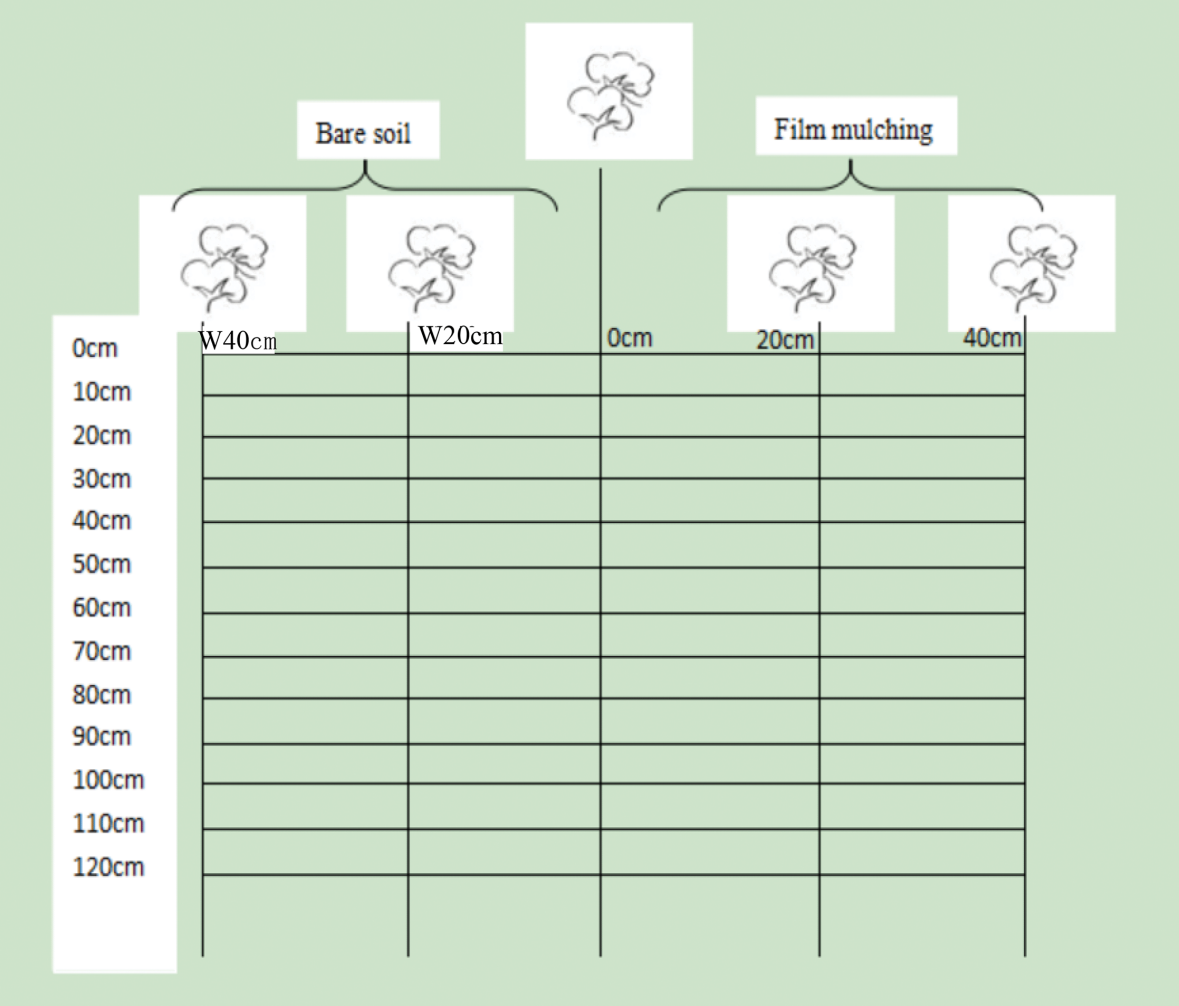
Diagrammatic representation of root sampling positions in 2012 and 2013. Note: Bareness at left part W20 cm and W40 cm yet film mulching at right part E20 cm and E40 cm, same as the following.

Root samples were collected once before each irrigation event (9 times) during each season. A soil core device (7 cm in diameter, 10 cm high) [34, 39–40] was used to obtain samples to minimize horizontal and vertical root damage. To study the horizontal distributions of the roots, root cores (soil samples containing roots) were collected in two directions (east and west) from the trunk region (0 cm) of the plants at distances of 20 and 40 cm, in which film mulch was applied in the east part (E20 and E40) yet bareness in the west part (W20 and W40) (Fig 1). The point furthest east of the trunk (E40 cm) was located in the center of the film, and the point furthest west of the trunk (W40 cm) was located in the center of the bare soil. To study the vertical root distribution, the auger was inserted to a depth of 120 cm from the soil surface. The resulting soil core was divided into 12 blocks of 0–10 cm, 10–20 cm, 20–30 cm, 30–40 cm, 40–50 cm, 50–60 cm, 60–70 cm, 70–80 cm, 80–90 cm, 90–100 cm, 100–110 cm and 110–120 cm in both sampling directions, and the soil was removed carefully using hand implements from each block to a depth of 10 cm. Simultaneously, each 10-cm-deep root core was placed in a 0.05 cm diameter circular grid mesh sieve and washed under running water to remove soil particles from the roots. Collected root samples were scanned with a scanner (Phantom 9800X, MiCROTEK, Shanghai, China) and analyzed using the DT-SCAN software (Delta-T Devices Ltd, UK) to determine the root length (RL), average root diameter and root surface area (RA). Next, the root length density (RLD, root length per volume of soil, mm/cm^3^) and root surface area density (RAD, root surface area per volume of soil, mm^2^/cm^3^) were determined as spatial distribution characteristics.

#### Root biomass

During sampling, at least two edge rows were excluded to avoid the boundary effects. After the leaf areas were measured, roots were sampled depending on their developmental stage every 15 days from May 15 to September 17 in 2012 and 2013, then dried in an oven at 80°C to a constant weight then weighed.

#### Yield and yield components

The seed cotton (SC) in each plot was harvested by hand three times every ten days from October 15, 2012 and October 10, 2013, and until October 20 to obtain the pre-frost seed cotton (PFSC). The lint yield (LY) and crack bolls were determined after ginning. The yield components included the total number of bolls (NB), the boll weight (BW) and lint percentage (LP: lint weight/seed cotton weight) ginned with a laboratory gin (MPSY-100A), which were determined by randomly harvesting 50 open bolls and weighing them after drying at each harvest.

### Statistics

The variations between the root sampling locations and years were considered to be random, the RLD and RAD data from two years were pooled together and interpolated using Kriging [41] to determine the two-dimensional root distributions in the different sampling growth stages. Meanwhile, a contour map was made using Surfer (Golden Software Inc., USA) to accurately describe the root development and distributions resulting from the different planting methods. Throughout all the growing stages, RAD and RLD (horizontal and vertical) were averaged to separately observe the variation of roots in both directions. To simulate the dynamics of RL and RA in the cotton fields mulched with film and investigate the relationships between RL, RA and cotton growth period (CGP), a non-linear regression analysis (cubic polynomial) was performed using CGP as the independent variable. The Logistic regression model was used to compare the RB and CGP for the different planting methods. Analysis of variance (ANOVA) was conducted using PROC GLM (Ver. 9.2, SAS institute Ltd., USA), which transplant system was a fixed effect. Duncan’s multiple testing at the 0.05 probability level was used to compare paired means. The data were checked and transformed to meet the ANOVA requirements (normal distribution, homogeneity of variance). Yield and yield components data were statistically analyzed using One-way ANOVA at a significance level of 0.05, with planting method as the independent variable and using Duncan’s Multiple Comparison Test to separate the means. OriginPro 8 (OriginLab) and Adobe Illustrator CS5 (Adobe) were used for plotting.

## Results

### The spatial distribution of cotton roots

#### Horizontal distribution of the cotton root systems

**Fig 2:**
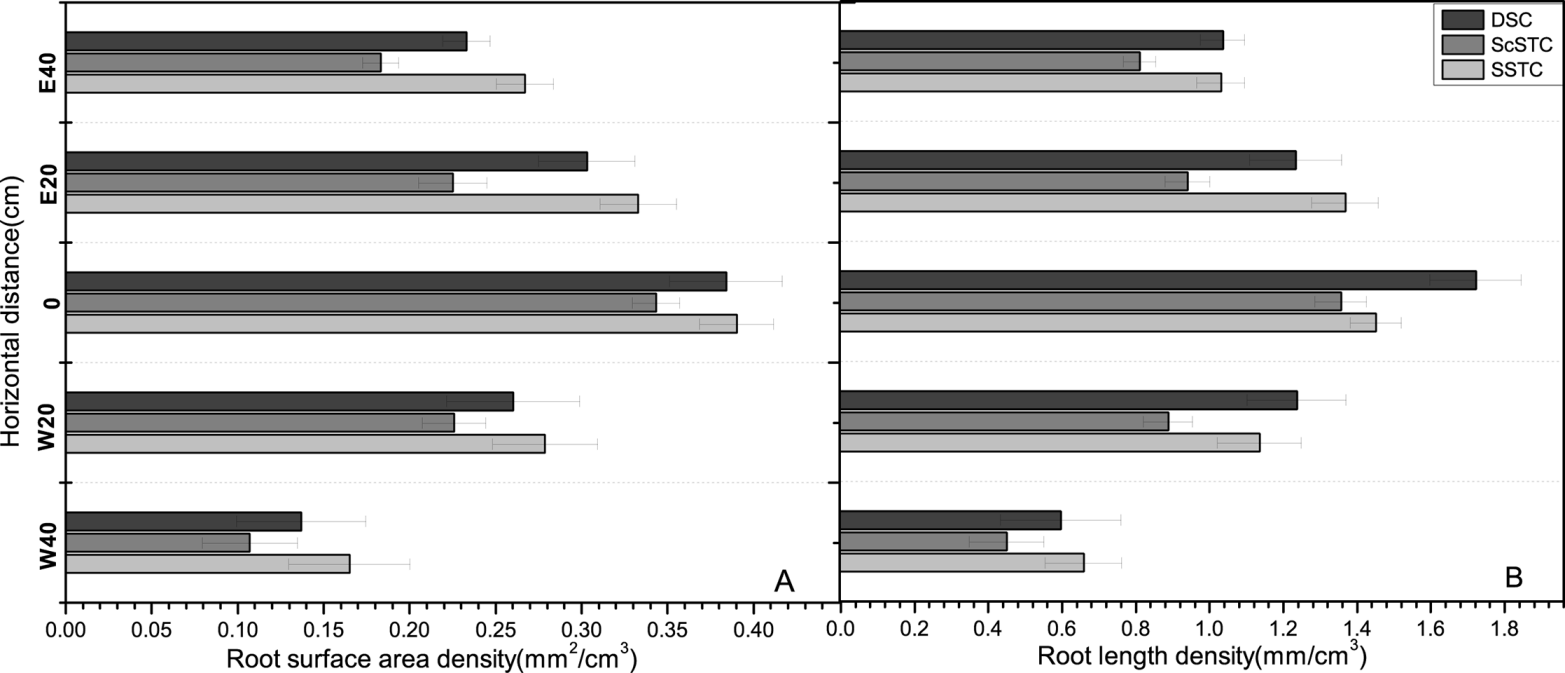
Horizontal distributions of RAD and RLD under planting systems of DSC, ScSTC and SSTC. Note: A and B represent the horizontal distributions of RAD and RLD in the different treatments, respectively.

Throughout all the growing stages, observations of the horizontal distributions of roots are presented in Fig 2. The RAD and RLD changed in the horizontal direction perpendicular to the plant rows in the three treatments. In general, the RAD and RLD decreased from the plant stalk to the west and east, with greater RADs and RLDs in the east (E20 cm and E40 cm) than in the west (W20 cm and W40 cm). The largest RADs and RLDs in the horizontal direction were found at 0 cm (where the taproots were), accounting for 24.77 and 24.11% of the total roots, respectively. The next largest RADs and RLDs were observed at E20 cm for film mulching, with values of approximately 23.76 and 23.58%, respectively. In addition, RADs and RLDs of 21.30 and 21.72%, 18.88 and 19.23%, and 11.28 and 11.35% occurred at W20 cm (bare soil), E40 cm (film mulched), and W40 cm (bare soil), respectively. The lowest values at W40 cm were attributed to the absence of film mulch which could increase the temperature of the soil to further promote root growth.

#### Vertical root distribution

**Fig 3:**
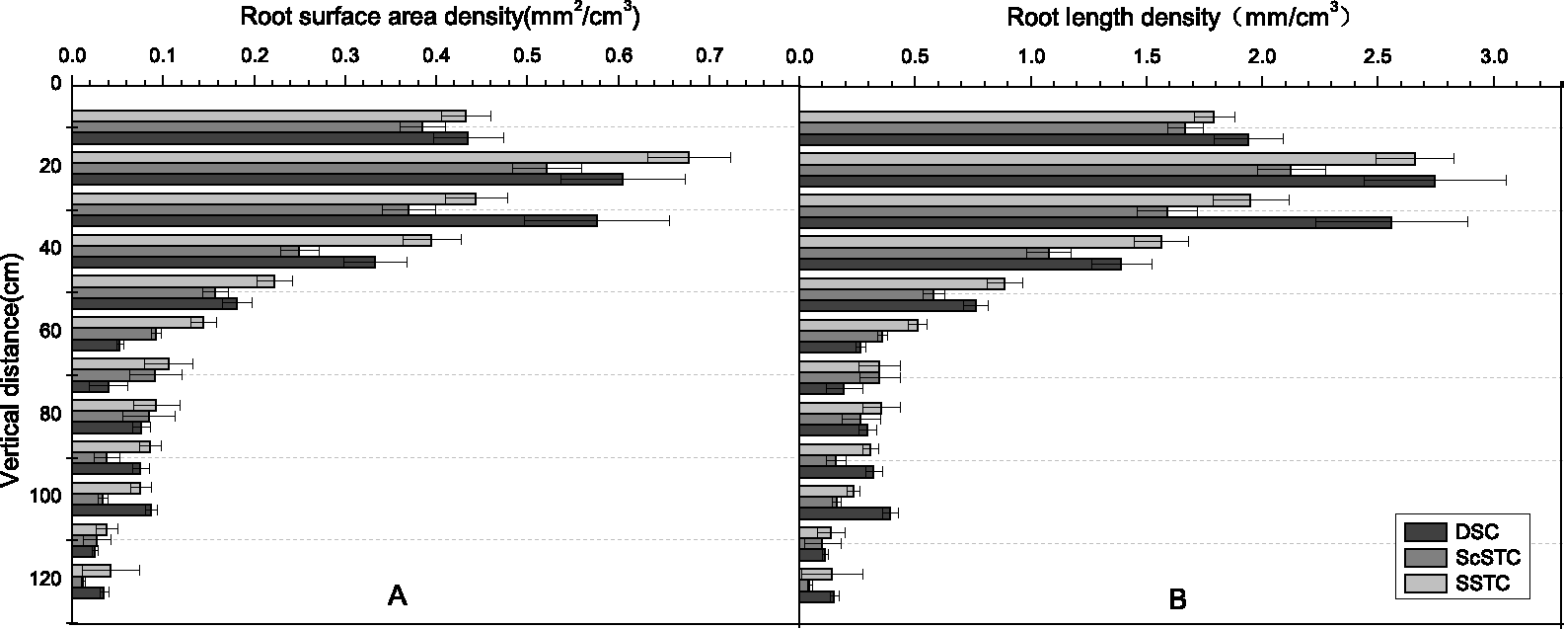
Vertical distributions of RAD and RLD under planting systems of DSC, ScSTC and SSTC. Note: A and B represent the horizontal distributions of RAD and RLD in the different treatments, respectively.

Throughout all the growing stages, the observed vertical root distributions are presented in Fig 3. Although the vertical root distribution pattern was uniform, significant differences were observed between the RADs and RLDs of the SSTC, ScSTC, and DSC with soil depth. The RADs and RLDs in the three treatments decreased with soil depth from 20 cm to 120 cm, and most RADs (0.68 mm^2^/cm^3^) and RLDs (2.66 mm/cm^3^) were observed at a depth of 20 cm in the SSTC. Additionally, 70% of total roots were located at depths of 0–40 cm, with the highest RAD (77.36%) and RLD (77.62%) occurring in the DSC, followed by the ScSTC (74.03% RAD and 76.25% RLD) and SSTC (70.75% RAD and 73.24% RLD), respectively. However, 13–20% of roots were produced at a depth of 40–80 cm, at which the RAD (20.51%) and RLD (19.26%) were greater in the SSTC than in the ScSTC (20.61% RAD and 18.30% RLD) and DSC (13.82% RAD and 13.61% RLD). Nevertheless, less than 10% of roots were located at depths of 80–120 cm, with RAD and RLD values of 8.82% and 8.77%, 8.74% and 7.50%, and 5.36% and 5.45% for the DSC, SSTC, and ScSTC, respectively.

#### Spatial distribution of the root length density (RLD) of the cotton transplanted from substrate (SSTC) in cotton seedling stage, boll stage and boll opening stage

**Fig 4:**
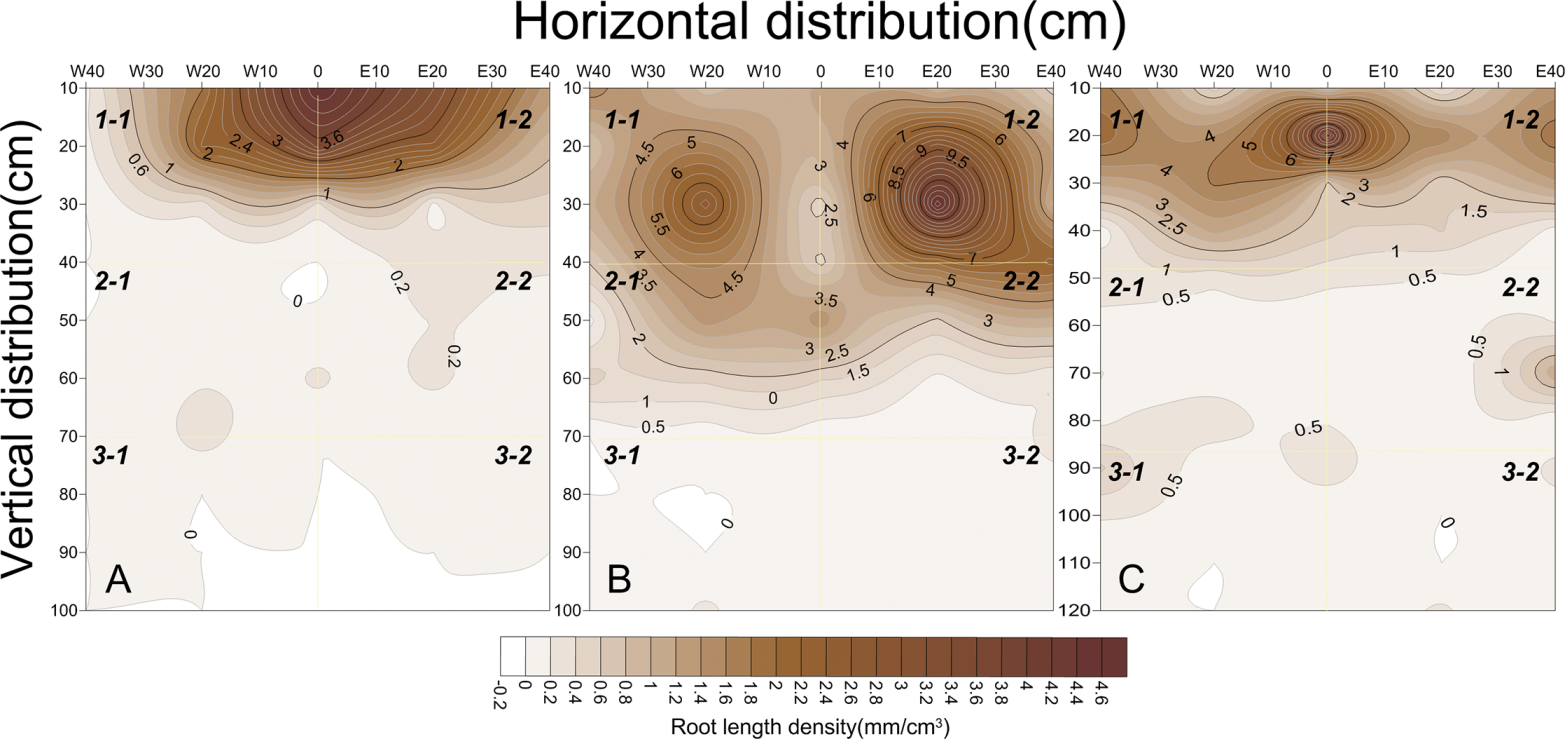
Spatial distributions of RLD under SSTC at cotton seedling stage, boll stage and boll opening stage. Note: A, B and C represented the seedling stage, boll stage and boll opening stage, respectively.

The RLDs were greatest at depths of 0–30 cm when the taproots grew vigorously (Fig 4A) and were lower at depths of 40–60 cm 46 days after transplanting (seedling stage). In addition, the RLD was greatest at 0 cm in the horizontal direction near the cotton taproots. However, the RLD was greater at E20 cm than at W20 cm because of the mulch film.

The roots reached their maximal lateral spread 91 days after transplanting (boll stage, Fig 4B), and many cotton roots were extended to depths of 0–50 cm depths. However, few roots grew to a depth of 60 cm. As lateral root growth was strong, the RLDs were greatest at E20 cm and W20 cm, which indicated that the roots systems were concentrated in the soil. Since the mulch film the soil temperature being increased, the RLD was significantly higher in the east (E20 cm and E40 cm) than in the west (W20 cm and W40 cm).

Similar RLD variations were observed at 125 days after transplanting (boll opening stage, Fig 4C). However, the lateral root density decreased sharply from the taproot. Additionally, fewer roots were found in the west than in the east, which was attributed to the vigorous growth of the cotton roots under the mulch film during the seedling stages.

#### Spatial distribution of the root length density (RLD) for the soil-cube seedling transplanted cotton (ScSTC) in cotton seedling stage, boll stage and boll opening stage

**Fig 5:**
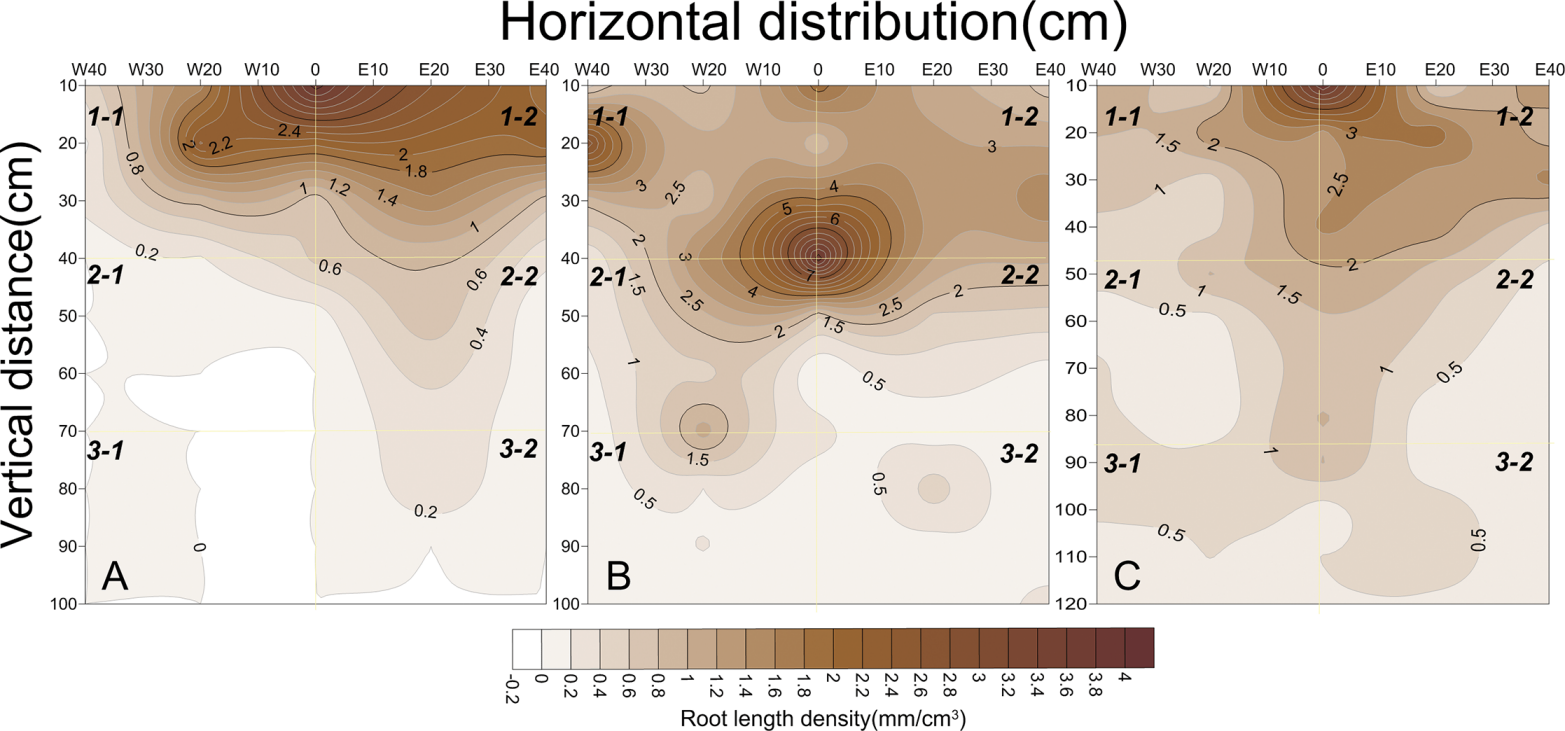
Spatial distributions of RLD under ScSTC at cotton seedling stage, boll stage and boll opening stage. Note: A, B and C represented the cotton seedling stage, boll stage and boll opening stage, respectively.

Differences in the RLDs in the ScSTC were consistent with those in the SSTC. The maximum RLDs were mainly distributed in the surface layer (0–30 cm) at 46 days after transplanting (seedling stage). However, the quantity of roots decreased sharply below a depth of 30 cm. Nevertheless, the roots penetrated to a depth of 60 cm and increased significantly at a depth of 30–50 cm at 91 days after transplanting (boll stage). Until 125 days after transplanting (boll opening stage), the RLD was greatest at depths of 0–50 cm but was lower than the RLD at 91 days after transplanting (Fig 5).

#### Spatial distribution of directly sown cotton (DSC) in cotton seedling stage, boll stage and boll opening stage

**Fig 6:**
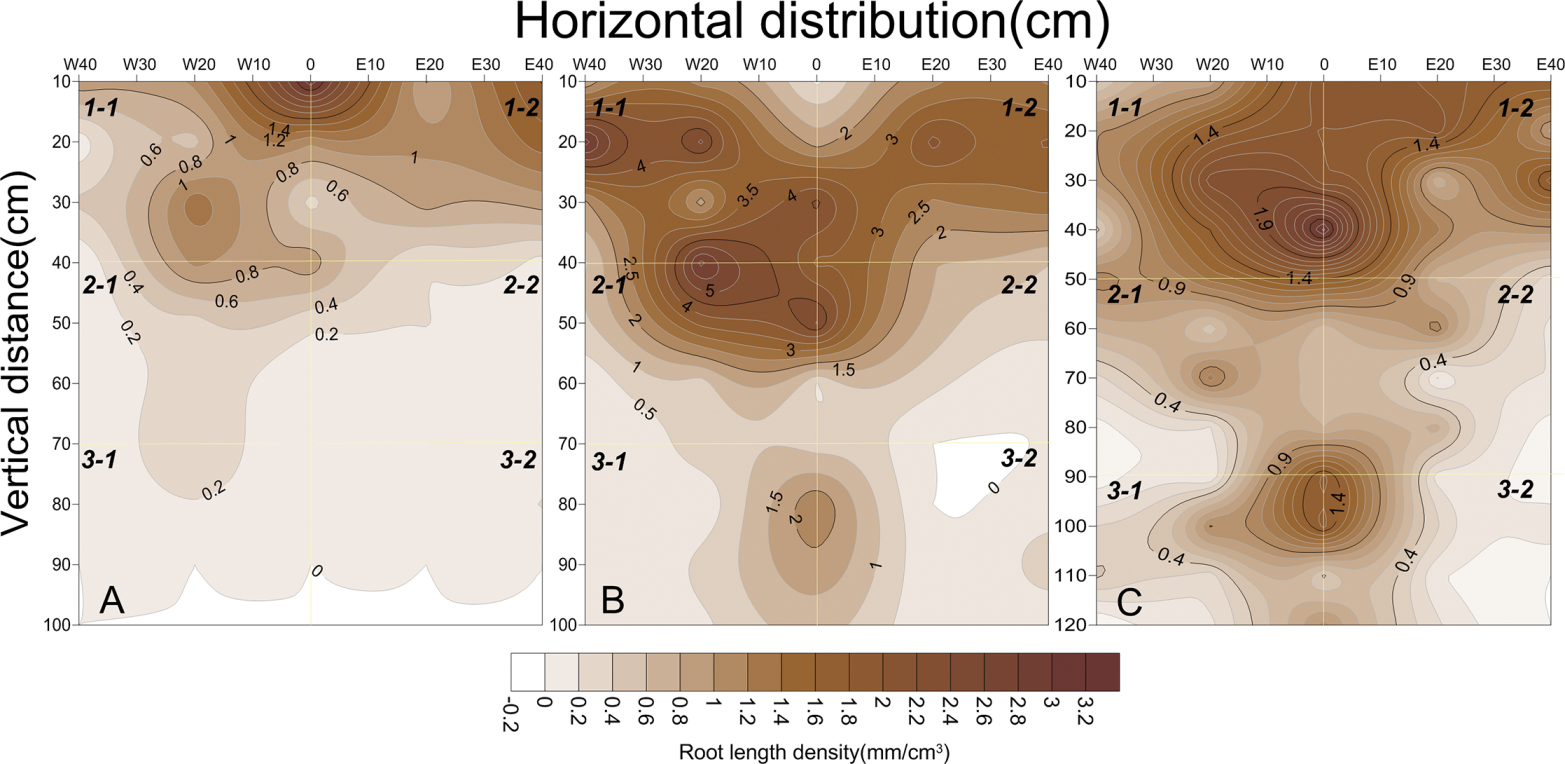
Spatial distributions of RLD under DSC at cotton seedling stage, boll stage and boll opening stage. Note: A, B and C represent the cotton seedling stage, boll stage and boll opening stage, respectively.

Similar varying tendency of RLDs under DSC, SSTC and ScSTC during all growing stages were consistent; however, the taproots of the SSTC and ScSTC only maintained exuberant growth on 15 June (seedling stage) and 30 July (boll stage). In addition, the taproots of the cotton in the DSC reached a depth of up to 100 cm in boll opening stage (Fig 6). While in seedling stage, most of the roots were located at depths of 0–30 cm, with few roots at depths of 40–60 cm. The RLD was greatest at a depth of 0–20 cm at the 0 cm location, which was consistent with SSTC and ScSTC.

#### Spatial root length density (RLD) variations in the different treatments

**Fig 7:**
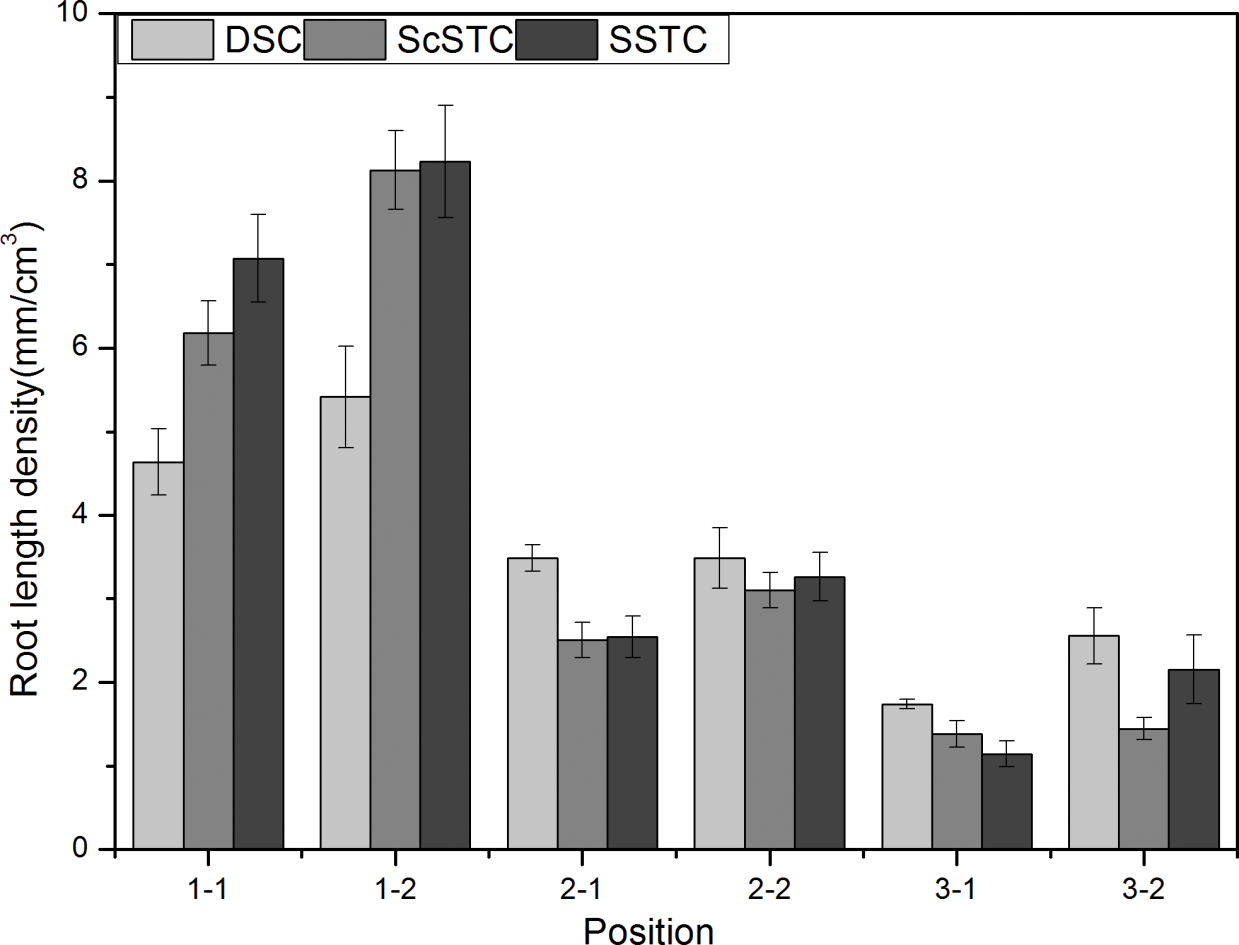
Variations in the accumulated RLDs at different spatial positions from DSC, ScSTC and SSTC.

The spatial distributions of the RLDs in the SSTC, ScSTC and DSC were divided into six equal parts (1–1, 1–2, 2–1, 2–2, 3–1 and 3–2) which were in the vertical direction from the bottom to the top and in the horizontal direction from left to right, respectively (Figs 4, 5 and 6). The average RLDs of the three treatments during the cotton growing stages at different points (1–1, 1–2, 2–1, 2–2, 3–1 and 3–2) are shown in Fig 7. Similar trend of RLD spatial distribution was found under SSTC, ScSTC and DSC, which indicated highest RLD occurred at the part of 1–2. In addition, greater RLDs were observed in 1–2 than in 1–1, for the reason of the film mulching in 1–2, similarly, RLD being higher at 2–2 and 3–2 comparing with 2–1 and 3–1 respectively. In 1–1 and 1–2, the RLDs under SSTC and ScSTC were significantly higher than the RLDs under DSC; however, no significant difference in RLDs was observed between SSTC and ScSTC. The RLDs of the DSC were highest than that of ScSTC and SSTC in 2–1, 2–2, 3–1 and 3–2, which indicated that the roots of DSCpenetrated deeper than the roots of SSTC and ScSTC. Additionally, the RLDs of the SSTC were higher than the RLDs of the ScSTC in 2–1, 2–2 and 3–2.

### Root surface area (RA) and root length (RL) dynamics

**Fig 8:**
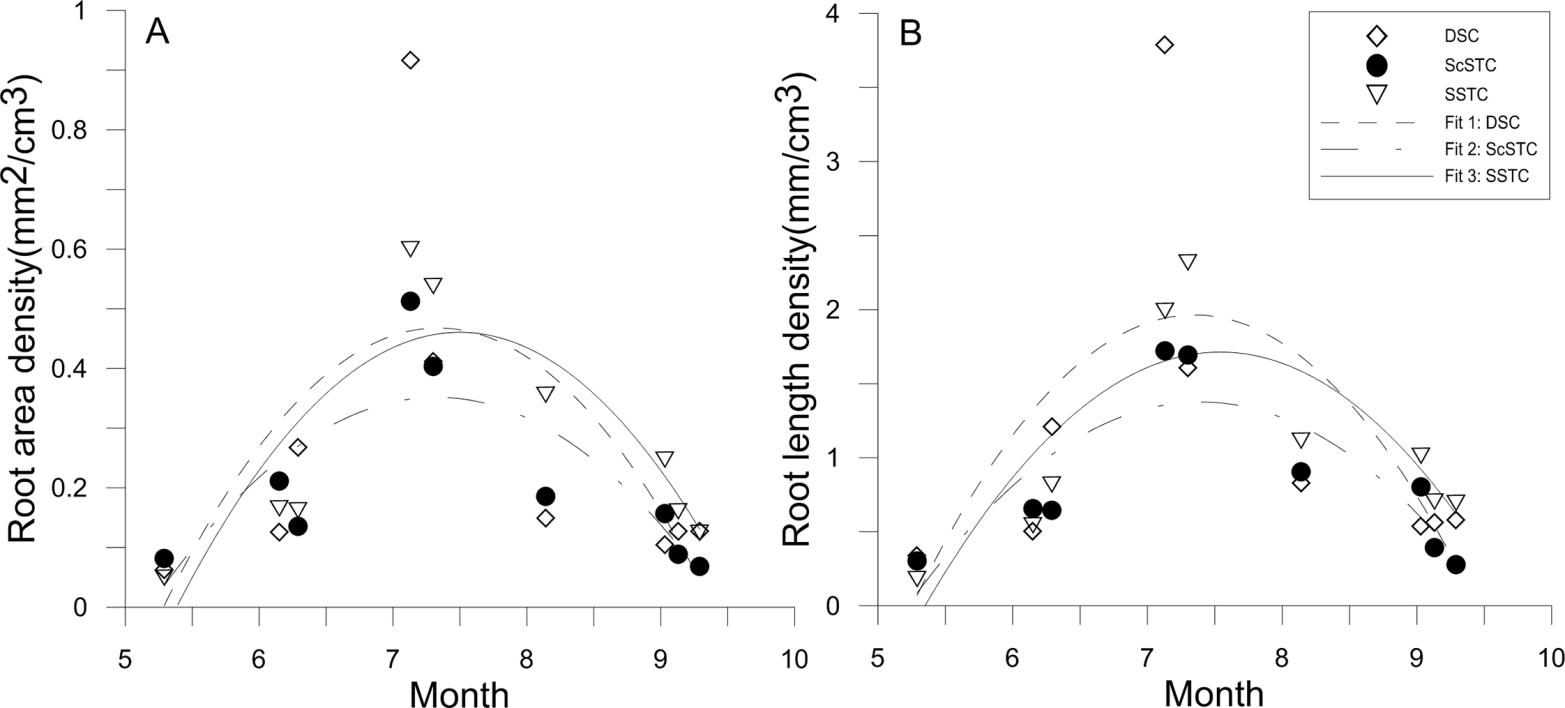
Variations of RAD and RLD in the different planting treatments over time.

The cubic functions for the RA and RL distributions in the SSTC, ScSTC and DSC obtained using regression analysis were shown in Table 1. The simulated RA and RL results agreed well with the measured results, as shown in Fig 8. The measured and simulated RA and RL continuously increased and sharply decreased, respectively, in mid-July in all treatments, and the maximum simulated RAs and RLs in the SSTC occurred 91–94 days after transplanting (August 1–4) and were 10370 mm^2^ plant^-1^ and 38486 mm plant^-1^, respectively. In the ScSTC, the maximum RA (7794 mm^2^ plant^-1^) and RL (31272 mm plant^-1^) occurred 85–87 days after transplanting (25–27 July). However, the highest RA (11102 mm^2^ plant^-1^) and RL (46942 mm plant^-1^) in the DSC occurred approximately 83 days after planting (15 July).

**Table 1:** Regression models of the surface area of cotton roots (Y_1_), cotton root length (Y_2_) and growth period (x) in the different treatments.

Treatments	Equation	R^2^	P Value
SSTC	Y_1_ = -10974.5607+471.1710X-2.7585X^2^+0.001719X1^3^	0.8732	0.0111
	Y_2_ = -51951.9515+2308.0153X-17.8969X^2^+0.037968X^3^	0.9077	0.0051
ScSTC	Y_1_ = -30096.4917+1519.4939X-10.6421X^2^+0.014764X^3^	0.7949	0.0359
	Y_2_ = -10562.4955+554.2102X-5.1191*X^2^+0.013215X^3^	0.9248	0.0031
DSC	Y_1_ = -197722.0337+7129.5754X-65.6171X^2^+0.183519X^3^	0.6257	0.1492
	Y_2_ = -49140.3785+1762.4553X-16.2732X^2^+0.045584X^3^	0.6244	0.1505

### Root biomass (RB) studies

In addition to the total RADs and RLDs in the different treatments, the RB was also measured at various cotton growth stages (Table 2). The dynamics of RB production in all treatments followed a typical sigmoid curve, with a lag phase followed by an exponential phase and a stationary phase. The accumulation of RB was significantly different between the treatments due to the duration of the exponential RB accumulation (Table 2). The SSTC and DSC generally had longer duration of exponential accumulation than the ScSTC (Table 2). However, no significant treatment difference in the maximal rate of exponential accumulation was observed. In the SSTC, the maximum accumulation rate of RB occurred from June 20–28. In addition, the RB value was maximized on July 4–9 and decreased on July 19, which indicated that the duration of exponential RB accumulation was prolonged to approximately 23–29 days. In the ScSTC, the maximum rate of RB accumulation occurred over four days, and in the SSTC, the maximum rate of RB accumulation occurred over 15–30 days. Moreover, in the DSC, the maximum rate of RB accumulation occurred from June 13–20, which was 6 days earlier than in the ScSTC.

**Table 2:** Eigenvalues of the accumulation of cotton root biomass under different planting patterns in 2012 and 2013.

Year	Equation		t_0_ ^†^(d)	t_1_ ^‡^(d)	t_2_^§^(d)	R^2^	P Value
2012	Y_SSTC_ = 18.19/(1+EXP(5.91–0.09X1))		65.89	51.21	80.58	0.9630	<0.001
	Y_ScSTC_ = 14.29/(1+EXP(5.799–0.09X1))		65.62	50.70	80.53	0.9670	<0.001
	Y_DSC_ = 17.71/(1+EXP(5.59–0.07X1))		83.93	64.15	103.70	0.9687	<0.001
2013	Y_SSTC_ = 16.05/(1+EXP(8.19–0.12X1))		70.44	59.12	81.76	0.9953	<0.001
	Y_ScSTC_ = 13.57/(1+EXP(10.93–0.17X1))		62.88	55.31	70.45	0.9763	<0.001
	Y_DSC_ = 14.60/(1+EXP(7.85–0.11X1))		68.61	57.09	80.12	0.9875	<0.001

† t_0_ is the day with the maximum RB accumulation

‡ t_1_ is the start date of the exponential period of RB accumulation

§ t_2_ is the last day of the exponential period of RB accumulation.

### Yield and yield components

The SSTC resulted in both 4.0% larger SC yields than the ScSTC and DSC in 2012 (Table 3). Significant differences in SC yield were observed in 2013, the SSTC had 8.0% and 5.2% higher SC yields than the ScSTC and DSC, respectively. Although there was no significant difference in LYs, the LYs were 1.7% and 1.1% greater in SSTC than in ScSTC and DSC in 2012, same being found in 2013 (7.2% and 2.3% larger). And no significant differences in BW and LP were observed among the treatments in 2012 and 2013. Furthermore, the PFSC in ScSTC was 1.3% greater than in SSTC and DSC in 2012, and 3.9% and 0.4% higher than PFSC in SSTC and DSC in 2013.

**Table 3:** Yield and yield components in 2012 and 2013.

Treatments	Yield/kg ha^-1^		PFSC/%	Yield components		
	SC	LY		NB/10000 ha^-1^	BW/g	LP/%
	2012					
SSTC	3693.13±34.29	1400.80±54.31	95.43±1.1	83.69±0.69	6.00±0.06	37.92±0.25
ScSTC	3546.24±138.62	1377.11±7.80	96.64±0.12	71.82±4.02	5.84±0.12	38.83±0.18
DSC	3547.20±141.65	1384.82±67.33	95.42±0.44	75.78±4.17	5.80±0.15	39.03±0.39
P value	0.0152	0.9441	0.0597	0.0720	0.5092	0.3147
	2013					
SSTC	3210.87±173.95	1328.88±72.05	95.12±0.77 B	69.19±3.44	6.29±0.04	41.39±0.26
ScSTC	2955.08±74.47	1233.25±30.36	98.92±0.25 A	58.80±1.18	6.08±0.09	41.74±0.14
DSC	3042.44±119.86	1298.92±52.02	98.49±0.18 A	62.63±2.32	6.10±0.04	42.69±0.15
P value	0.0389	0.4732	0.0006	0.0429	0.0782	0.3400

Data represent mean ± SD for at least 3–4 sets of observations, A and B indicate a significant difference at the 0.01 level.

## Discussion

### The spatial distribution of cotton roots

The roots of any plant play a pivotal role in the plants life cycle [42]. Cotton has a deep root system that is helpful for up-taking water and nutrients from deeper soil layers. Knowledge of the root distribution patterns of labor-saving seedling transplanting methods can be used for nutrient and irrigation management in cotton cultivation practices.

The influences of SSTC on cotton root development could be better understood by describing their spatial distribution [30]. The maximum horizontal root spread in SSTC was 0-E20 cm from the cotton row. This pattern is mainly attributed to the reduced soil evaporation and increased soil temperature due to plastic film mulching after transplanting [3, 43]. In addition, being consistent with previous studies [34, 44–45], the RADs and RLDs in all treatments were greater at E20 cm than at W20 cm in this study. Moreover, the quantity of cotton roots under the film mulch (E20 cm and E40 cm) increased by 8–12% compared with that of the bare soil (W20 cm and W40 cm).

The differences in the RADs and RLDs in the vertical direction were significant at all of the studied depths. The decrease of RLD with depth could be attributed to root growth begins in the top soil layer, thus roots grow for a longer time in the top soil layer than in deeper soil layers. Consequently, the RB in the surface layers would be greater before the roots grew to deeper depths [46]. In this study, the curves of RAD and RLD were the typical unimodal sort, with most roots concentrated at depths of 20–40 depths, as previous reported [42, 47–49]. In addition, the maximum rooting depths were 20–40 in all treatments, and the rooting depths in the DSC were approximately 10 cm greater than those in the SSTC and ScSTC, potentially because transplanting disturbs root growth [16]. However, in this study, the RADs and RLDs were significantly different between SSTC and DSC in both horizontal and vertical directions.

### Root development during the cotton growth stages

The physical arrangement of the roots greatly affects a plants ability to capture nutrients and water resources, consequently, could be used to determine whether cotton will flourish or perish. Therefore, cotton root growth was initiated at the cotton seedling stage, followed by vigorous development during the cotton boll stage and slower development during the cotton boll opening stage [10]. Previous studies have shown RLDs being 0.2–0.3 cm/cm^3^ are satisfactory for cotton growth [50]. In this study, the maximum RLD per plant was 2.66 mm/cm^3^ at a depth of 10–20 cm depths. The SSTC provided a fairly high RLD which met the needs for cotton development. Moreover, the maximum rate of root growth in the SSTC occurred 91–94 days after transplanting (August 1–4), and the maximum rate of root growth in the ScSTC occurred 83 days after transplanting. These results indicated that early root development occurred in the ScSTC, which could explain the higher PFSC in ScSTC than in SSTC. However, according to Fig 8, the RAD and RLD in the ScSTC were lower than the RAD and RLD in the SSTC after the cotton squaring stage. Moreover, both RAD and RLD in the SSTC were greatest after the cotton boll opening stage. All in all, vigorous root development in the SSTC occurred at the key cotton growing stages, which is of great significance, though late-onset root growth.

### Root biomass (RB) and yield

Similarly, early onset root growth in ScSTC was observed, and the RB varied during the different growing stages. The final dry weight of the cotton roots was mainly controlled by the duration of the exponential accumulation of RB [51]. This study, demonstrated SC of SSTC was 4.0% (2012) and 9.0% (2013). In addition, lateral root growth was governed by the current root mass or RLD and the soil water availability at a given depth [46], which could also explain the greater number of lateral roots in the SSTC (Fig 4; Fig 5). More lateral roots could also result from the looser substrate texture in the SSTC than in the ScSTC because compacted soils generally provide greater mechanical constraints than loose soils and the transport of oxygen and CO_2_ to and from the plant roots, respectively, are reduced in compact soils [52]. Furthermore, previous studies have reported that roots without branches generally have a shorter lifespan than roots with branches [53].

Increased RL and RLD also improved plant root anchorage [52]. Cultural practices based on the establishment of root systems that extend the flowering period or enhance plant growth and development, such as planting cotton early, likely increase crop productivity [54]. Transplanting seedlings to avoid early season chilling stress could be achieved by using greenhouse-like huts. Moreover, previous studies have reported that the blooming period was extended by approximately one week and that peak blooming occurred 5 days earlier in transplanted cotton than in DSC [3]. Several studies have indicated that increased crop yields can be attributed to changes in root architecture [55]. Additionally, the results in this study indicated that the SSTC had significant increases in SC yields in 2012 and 2013. Thus, the environment was suitable for the cotton growth stages and high SC yields were maintained in the SSTC, which can be explained by more NB and greater BW in SSTC than in ScSTC and DSC.

## Conclusions

Data from the two field experiment seasons were used to investigate the RAD and RLD distributions resulting from different seedling transplanting methods. Horizontally, the cotton RAD and RLD were greatest at 0 cm and were greater closer to the cotton plants and under the mulch film. Vertically, the RADs and RLDs in the different treatments decreased with depth and increased with time. The SSTC resulted in higher RADs and RLDs in the horizontal and vertical directions, followed by the DSC and ScSTC.

Firstly, the strongest root growth occurred during the cotton flowering and boll stages in the SSTC, with early onset root growth in the ScSTC and vigorous root growth throughout all of the cotton growth stages in the DSC. Secondly, longer exponential increases of RB accumulation resulted in higher SC yields. Thirdly, the highest PFSC occurred in ScSTC in 2012 and 2013, these enhancements were mainly attributed to the early onset root growth pattern in the ScSTC.

Understanding the root dynamics and yield variances of cotton resulting from different planting methods is helpful for promoting the use of cotton seedling nurseries to improve plant performance and alleviate the growing problem of labor shortage. In this study, progress was made regarding understating the yield and labor-saving advantages of transplanting cotton seedlings.

## Supporting information

S1 File Data of roots spatial distribution and volume. (ZIP)
